# Species-specific qPCR assays allow for high-resolution population assessment of four species avian schistosome that cause swimmer's itch in recreational lakes

**DOI:** 10.1016/j.ijppaw.2019.04.006

**Published:** 2019-04-22

**Authors:** Sydney P. Rudko, Alyssa Turnbull, Ronald L. Reimink, Kelsey Froelich, Patrick C. Hanington

**Affiliations:** a 357 South Academic Building, School of Public Health, University of Alberta, 116st and 85 Ave, Edmonton, Alberta, Canada, T6G 2R3; b Office of Campus Ministries, 129 E. 10th St. Hope College, Holland, MI, 49423, USA; c Freshwater Solutions LLC, 6906 48th Ave, Hudsonville, MI, 49426, USA; d Saint Joseph High School, 2521 Stadium Dr., Saint Joseph, MI, 49085, USA

**Keywords:** qPCR, Trematodes, Swimmer's itch, Environmental transmission, Recreational water, Trichobilharzia, Schistosoma, eDNA

## Abstract

Swimmer's itch is an allergic condition that occurs when the motile and infectious stage of avian schistosomes penetrate the skin of an individual. Flatworm parasites that cause swimmer's itch belong to the family Schistosomatidae. They utilize a variety of different species of bird and mammal as definitive hosts, and rely on different species of snail, in which they complete their larval development to culminate in a motile, aquatic, infectious stage called a cercaria. Recently, qPCR-based assays have been developed to monitor for swimmer's itch-causing trematodes in recreational water. This environmental DNA approach has been useful for quantifying the abundance of the free-living cercaria, the causative agent of swimmer's itch. However, the existing qPCR test amplifies from all known schistosome species, making it excellent for assessing a site for swimmer's itch potential, but not useful in determining the specific species contributing to swimmer's itch or the likely hosts (snail and bird) of the swimmer's itch-causing parasites. Thus, species-specific resolution built into a qPCR test would be useful in answering ecological questions about swimmer's itch cause, and efficacy of control efforts. This paper details bird, snail, and cercaria surveys conducted in the summer of 2018, that culminated in the development and deployment of four species-specific qPCR assays, capable of detecting *Trichobilharzia stagnicolae, Trichobilharzia szidati, Trichobilharzia physellae, and Anserobilharzia brantae* in recreational water. These assays were used to assess the relative abundance of each parasite in water samples collected from lakes in Northern Michigan.

## Highlights

• qPCR diagnostic tests of four species of avian schistosomes.
• Comprehensive snail, bird, and cercariae surveys in Northern Michigan.
• Use of qPCR cercariometry in water to measure swimmer's itch.

## Graphical abstract

**Figure:**
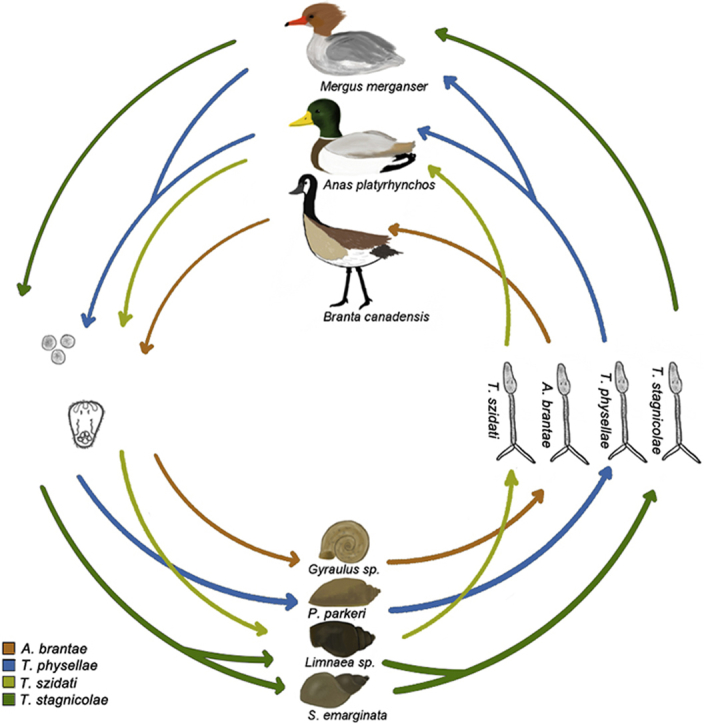

## 1 Introduction

1To complete their life cycle, trematodes from the family Schistosomatidae utilize primarily avian or, mammalian (often rodent) definitive hosts and snail intermediate hosts (Brant and Loker, 2005; Gordy et al., 2018; Brant and Loker, 2009a). Embryonated eggs leave the definitive host, hatch into free-living miracidia, which actively seek out a snail host. Upon finding a snail, they penetrate the snail intermediate host. Inside the snail, miracidia develop into sporocysts, which produce cercariae asexually. The free-living cercariae are then shed from the snail host back into the water where they search for their definitive host. Accidental contact and penetration into human skin may cause the allergic condition known as cercarial dermatitis or swimmer's itch. Trematodes of the genera *Trichobilharzia, Gigantobilharzia, Dendritobilharzia, and Schistosomatium* have all been implicated in swimmer's itch outbreaks (Baird and Wear, 1987; Batten, 1956; Coady et al., 2006; Cort, 1928; Kolářová et al., 1999; Verbrugge et al., 2004; Zbikowska, 2004).

Numerous species of schistosome are capable of causing swimmer's itch in Michigan. Past studies have found that *T. stagnicolae* is very common in the region, cycling through *Stagnicola emgarinata* snails as the intermediate host, and common mergansers as the avian definitive host (*Mergus merganser*). As part of a previous study, we also confirmed *Dendritobilharzia* sp. Miracidia in the feces of a mallard duck (*Anas platyrhynchos*) and a Canada goose (*Branta canadensis*) (Rudko et al., 2018). Others have reported *Gigantobilharzia huronensis* cycling in red winged blackbirds, as well as *T. szidati* and *T. elvae* in *Lymnaea stagnalis* snails (Strohmt et al., 1981).

Removing summer resident common mergansers was shown to be an effective swimmer's itch control method on Glen Lake in Leelanau County, MI, (Blankespoor and Reimink, 1991). This methodology continued on similar lakes in Michigan and Maine through the 1990s and early 2000s, and again beginning in 2015. In 2018, the Michigan Department of Natural Resources began providing nuisance control permits to trap and relocate summer resident common mergansers for lakes that can demonstrate there is a swimmer's itch problem caused, at least in part, by *T. stagnicolae* cycling through *S. emarginata* and common mergansers. Our fieldwork in 2017 and 2018, along with reports from residents at specific lakes, showed that despite extensive control efforts on a number of lakes in Northern Michigan, some programs have not been effective at reducing the snail infection prevalence or the number of schistosome cercariae in the water (Reimink and Hanington, 2017). To understand why merganser relocation was unsuccessful, improved resolution in swimmer's itch assessment tools was required.

The utility of PCR as a method for the detection of avian schistosomes in surface water samples was first demonstrated Schets et al. (2010), utilizing an assay developed by Hertel et al. (2002). More recently in 2013, Kane et al., 2013 demonstrated the detection and quantification of schistosome DNA in snails using real-time PCR, which demonstrates the flexibility of molecular detection methods in parasitology. Finally, in 2015 a pan-avian trematode qPCR assay, capable of detecting all members of the family Schistosomatidae was developed (Narayanan et al., 2015), and has been utilized as a DNA-based method for assessing cercariae abundance at recreational beaches (Rudko et al., 2018; Froelich et al., 2019). It has also been used to advance our understanding of how the physical environment plays a role in concentrating cercariae under certain conditions (notably wind direction) (Rudko et al., 2018). This test has been particularly useful in measuring the relative abundance of avian schistosomes in the lakes of Michigan, where swimmer's itch is a significant recreational water issue (Rudko et al., 2018).

Quantitative polymerase chain reaction (qPCR) exhibits incredible sensitivity and specificity, with the ability to accurately amplify DNA of specific species of parasite from environmental water samples. Water sampling and detection of environmental DNA (eDNA) is faster than snail collection and analysis, and provides more consistent data since representative snail sampling can be problematic due to low infection prevalence and uneven host distribution (Bass et al., 2015; Brown et al., 1988; Crews and Esch, 1986; Coady et al., 2006). Molecular methods are more cost effective and less labor intensive than specimen collection (Huver et al., 2015). With sufficient genetic information, species-specific qPCR tests can be developed, which would allow for assessment of multiple avian schistosome populations within a complex water sample. This is akin to the approach used in Hust et al. (2004), which used the ITS region to discriminate between two microphallid species in their intermediate host. Various applications of eDNA testing to environmental parasitology have been recently reviewed in Bass et al. (2015).

The purpose of this study was two-fold. First we report four new diagnostic tests to detect the swimmer's itch causing parasites in recreational water and in doing so demonstrate that detection of a specific schistosome species from a complex sample is possible. Secondly, we deployed these tests to attempt to determine why swimmer's itch continues to persist in Northern Michigan despite an extensive control program. The four qPCR assays developed in this study are specific to the cytochrome oxidase C gene (CO1) of three species of *Trichobilharzia,* and a species of *Anserobilharzia* in Northern Michigan. These species were selected because snail surveys conducted in 2018 on numerous lakes in Michigan found them to be the most prevalent swimmer's itch-causing parasites in the area. Each species cycles through a unique bird host, and utilizes snails common to all lakes in the region. These assays were deployed to determine the relative abundance of each species in water samples collected as part of a swimmer's itch monitoring program in Northern Michigan in 2018. Snail and waterfowl diversity and schistosome diversity, as measured by CO1 barcoding of miracidia and cercariae, was determined for each lake. Cercariae abundance in each water sample was assessed using the pan-avian schistosome qPCR test (Narayanan et al., 2015; Rudko et al., 2018), and a semi-quantitative assessment of the avian schistosome species found in those water samples testing positive using the pan-avian schistosome assay was conducted using the species-specific qPCR assays reported in this paper.

## 2 Methods

### 2.1 Locations

Seven lakes in Northern Michigan were selected for study: Big and Little Glen (44.868116, - 85.959403), Lime (44.895839, -85.840707), North (45.028172, - 85.725632) and South (44.875921, - 85.711454) Lake Leelanau (Leelanau County); Long, Elk and Skegemog (44.866540, - 85.380531) Lakes (Grand Traverse County); Charlevoix (45.269175, -85.128350), Walloon (45.279333, - 85.007122) Lake (Charlevoix County). The lakes monitored were selected because they had either been removing and relocating common mergansers for the last 1–3 years, were attempting to qualify for a trap and relocation permit, or were simply interested in conducting a full assessment to determine the extent of their problem.

### 2.2 Snail and larval trematode collection and processing

**Fig. 1:**
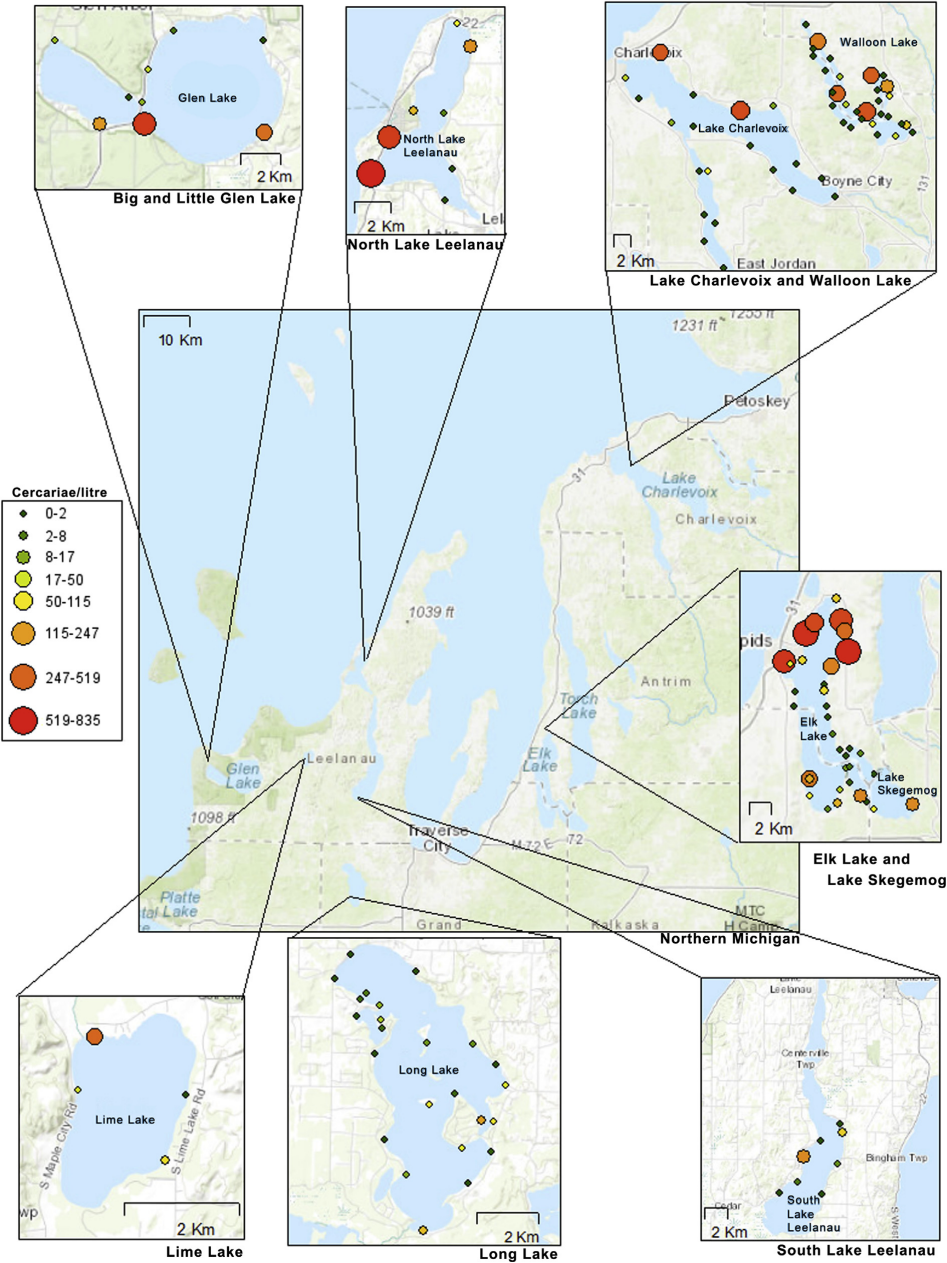
**Abundance of cercariae by sampling site**. Water samples were obtained in mid-June and cercariae abundance was determined using the pan-avian schistosomes qPCR.

To collect snails, 1 m^2^ weighted hoops were randomly tossed throughout each shoreline collection site (collection sites can be seen in Fig. 1). Collection sites were selected with the help of local riparians and were places where people reported contracting swimmer's itch. All snails within the hoops were collected using specially designed snail scoops with attached mesh bag nets. Following collection, snails were isolated into individual plastic wells filled with well water, housed throughout the day and night, and exposed to bright natural and fluorescent light the next morning, as described in Blankespoor and Reimink (1991). Snail genera were identified morphologically. Trematode cercariae shed from snails were pipetted into collection tubes and preserved with 95% ethanol (schistosomes were noted). Ethanol preserved samples were stored at ∼ −20C and then transported to the University of Alberta for further processing. DNA extraction, PCR amplification of Cytochrome *c* oxidase 1 (CO1) gene, Sanger sequencing, and alignments were completed as described in Gordy et al. (2018).

### 2.3 Bird surveys

Diversity of the avian community within each lake ecosystem was measured by conducting a complete shoreline waterfowl survey, traversing the near-shore perimeter slowly by boat. All waterfowl were observed with the aid of field glasses and recorded by species, location, sex, and age (hatch-year and after hatch-year). Whole-lake surveys were conducted in mid-July when most broods had hatched yet had not begun flying.

### 2.4 Water sampling

Water sampling was performed according to Rudko et al. (2018). Briefly, a plankton tow was used to concentrate 25 1-L aliquots of water from each sampling location. Concentrated samples were then filtered again through a 0.4 μM filter (Pall FML 1050) and DNA extracted from the filters.

### 2.5 DNA extraction from cercariae

Ethanol (95%) preserved cercariae were extracted by first removing the ethanol using a vacuum centrifuge. Once dried, DNA extraction was performed as described in Rudko et al. (2018) using the Qiagen DNAeasy Blood and Tissues kit. The standard tissue lysis protocol was followed, but only using 25% of the buffer volume recommend in the protocol.

### 2.6 Extraction of schistosome DNA from water samples

DNA extraction from water samples was performed as described in Rudko et al. (2018) using the Qiagen DNAeasy Blood and Tissues kit. Briefly, filters were bead beat using a Beadmill homogenizer for 2 min at 3.1 m/s before continuing with the DNAeasy protocol.

### 2.7 DNA barcoding

The CO1 region was PCR amplified from the DNA extracted from purified cercariae/miracidia samples using primers CO1F15 and CO1R15 (Table 1) (Brant and Loker, 2009b). PCR products were Sanger sequenced (Macrogen Inc, South Korea). Forward and reverse sequences were trimmed prior to being pairwise aligned using Geneious.

**Table 1:** Primers and Probes. Asterisks (*) indicate reported in this paper, plus signs (+) indicate a locked nucleic acid at the preceding nucleotide position.

Primer Name	Sequence (5′-3′)	Reference
JVSF 18S	AGCCTTTCAGCCGTATCTGT	Narayanan et al. (2015)
JVSP 18S	/FAM/AGGCC/ZEN/TGCCTTGAGCACT/IABkFQ/	Narayanan et al. (2015)
JVSR 18S	TCGGGAGCGGACGGCATCTTTA	Narayanan et al. (2015)
CO1R15	TGAGCWAYHACAAAYCAHGTATC	(Brant and Loker, 2009a,b)
CO1F15	TTTNTYTCTTTRGATCATAAGC	(Brant and Loker, 2009a,b)
SRTP FWD	TGGTTTGGTWTGTGCTATGGG	*
SRTP PRB	/FAM/TGAGC + TCA + TACTACACTACC + TAAAC/IABkFQ/	*
SRTP REV	AKTCTTAACATCCAATCCY	*
SRTS FWD	ATTATCTAATTACTAATCATGGGATTGCA	*
SRTS PRB	/FAM/ACCAAACCC/ZEN/ACCAATCAATACAGGCA/IABkFQ/	*
SRTS REV	ATGCCAAATCATCTAAACCCAAC	*
SRTSZ FWD	GTTGTTGGGTTCTGTTAAATTTATAAC	*
SRSZ PRB	/FAM/TCTTAGTTC/ZEN/TCGGGTTTCGGTTGTTGTT/IABkFQ/	*
SRSZ REV	AGACGTAAACAAATACGCCCA	*
SRAB FWD	GATTCCTTCAGAGATTTATAAATATTTA	*
SRAB PRB	/FAM/TACCAAACC/ZEN/CRCCAATRAACACRGGCA/IABkFQ/	*
SRAB REV	ACGAGGTAACGCCAAATC	*

FAM = fluorescein; IABkFQ = Iowa Black fluorescent quencher.

### 2.8 Design and validation of the species-specific qPCR assays

Species-specific qPCR assays (primers and Taqman probe) were designed specific to the CO1 genes *of T. stagnicolae, T. szidati (* = *T. ocellaea), T. physellae,* and *Anserobilharzia brantae (* = *T. brantae).* These species were selected because they were most prevalent at the lakes surveyed (Table 1, Supplementary Fig. 1). Primers and probe SRTP FWD, REV and PRB, which are listed in Table 1were developed to detect First an *in silico* alignment analysis of the CO1 region was conducted to find appropriate regions to target for diagnostic testing (Supplementary Fig. 1). These regions were cross-referenced against sequences from related avian schistosome species to ensure cross reactivity would not occur and to identify unique regions to each species that could serve as targets for qPCR primers/probes. Selected assays were characterized and validated using plasmid standards containing the targets of interest and were also validated against the DNA of purified cercariae (avian and non-avian schistosomes), to confirm that there was no cross reactivity.

### 2.9 Avian schistosome qPCR and species-specific qPCR

The 18S pan-avian schistosome qPCR was carried out as described in Rudko et al., (2018). To calculate absolute numbers of cercariae based on the amount of DNA detected, we performed the method from Rudko et al., (2018), briefly a percent recovery of 4% was assumed, and the formulae *x = *[(*y* + 56521)/57736]. The species-specific qPCR assays were performed as follows; 200 nm each of primer and probe were added to Prime Time Gene Expression Master Mix (IDT) in a clean room. Samples were run in duplicate and copy numbers were ascertained for each run by performing a standard curve consisting of 50,000, 5,000, 500, 50, 5 and 0.5 copies of purified plasmid DNA cloned with the CO1 gene of each respective species. Genes were synthesized by IDT, and cloned into pJET 1.2. Fast cycling was performed on the Quantstudio 3 (Applied Biosystems), using the following protocol: 95**°** hold (20 s), 95**°** denaturation (1 s), 60**°** annealing (20 s).

### 2.10 Data analysis

Calculations (i.e.: cercariae abundance and waterfowl density) were performed in Microsoft Excel, and heat maps were made in ArcMap (10.6).

## 3 Results

### 3.1 Survey results

Between June and August of 2018, 11,309 snails were collected from the lakes in Northern Michigan. Each snail was visually identified to the genus level. Each snail was then isolated and monitored over the course of ∼24 h for parasite shedding. Cercariae that were putatively identified as schistosomes based on gross morphology (ie: eyespots and a bifurcated tail) and the observation of phototactic behavior (27 in total) were characterized using CO1 barcoding. Miracidia samples collected from fresh feces from hatch-year waterfowl (to ensure the infection was contracted on the sampled lake) were also sequenced via the CO1 region. From these results we identified *T. physellae* (shed from *Physa parkeri), T. stagnicolae* (*Stagnicola emarginata), T. szidati* (*Lymnaea* sp.*),* and *A. brantae (Gyralus* sp.) (Fig. 3). Additionally, we found miracidia from both *T. stagnicolae* and *T. physellae* in the feces of common mergansers, and found *T. physellae* and an unknown member of the Schistosomatidae family miracidia in the feces of mallards. (Table 2). *T. stagnicolae* was the most common species of cercariae identified. Nucleotide sequences reported in this paper have been deposited into Genbank under Genbank accession numbers **MK433243, MK433244, MK433245, MK433246, MK433247, MK433248, MK433249, MK433250, MK433251 MK433252.**

**Table 2:** Species of avian schistosomes barcoded and identified in this paper.

Sample ID	Lake	Host	Species	% Identity
Miracidia Samples				
ATSR1	Walloon Lake	Common Merganser	*T. physellae*	99.9
ATSR4	Long Lake	Mallard	Avian schistosomatid	87.6
ATSR5	Long Lake	Mallard	Avian schistosomatid	87.4
ATSR6	Long Lake	Mallard	Avian schistosomatid	87.6
ATSR7	South Lake Leelanau	Mallard	Avian schistosomatid	87.9
ATSR11	Lake Charlevoix	Common Merganser	*T. physellae*	99.6
ATSR12	Lake Charlevoix	Common Merganser	*T. physellae*	99.7
ATSR14	Lake Charlevoix	Common Merganser	*T. physellae*	100
ATSR15	Lake Charlevoix	Mallard	*T. physellae*	99.8
ATSR17	Glen Lake	Mallard	Avian schistosomatid	87.1
ATSR19	Glen Lake	Common Merganser	*T. stagnicolae*	99.6
ATSR20	Glen Lake	Common Merganser	*T. stagnicolae*	98
ATSR21	Elk Lake	Common Merganser	*Trichobilharzia* sp.	92.9
ATSR22	Elk Lake	Common Merganser	*T. stagnicolae*	99.4
ATSR23	Elk Lake	Common Merganser	*T. stagnicolae*	100
Cercariae Samples				
ATSR106	South Lake Leelanau	*P. parkeri*	*T. physellae*	99.9
ATSR179	Walloon Lake	*S. emarginata*	*T. stagnicolae*	99.9
ATSR180	Walloon Lake	*S. emarginata*	*T. stagnicolae*	99.8
ATSR249	Elk Lake	*S. emarginata*	*T. stagnicolae*	99.1
ATSR250	Elk Lake	*S. emarginata*	*T. stagnicolae*	98.4
ATSR253	Elk Lake	*S. emarginata*	*T. stagnicolae*	99.7
ATSR848	Lime Lake	*Gyraulus* sp.	*A. brantae*	99.3
ATSR851	Lime Lake	*Lymnaea* sp.	*T. szidati*	99.5
ATSR853	Lime Lake	*Lymnaea* sp.	*T. stagnicolae*	99.7
ATSR854	Lime Lake	*Gyraulus* sp.	*A. brantae*	99.5
ATSR974	Lake Charlevoix	*P. parkeri*	*T. physellae*	99.5
ATSR1024	North Lake Leelanau	*S. emarginata*	*T. stagnicolae*	99.6
ATSR1186	North Lake Leelanau	*S. emarginata*	*T. stagnicolae*	99.8
ATSR1358	North Lake Leelanau	*S. emarginata*	*T. stagnicolae*	99.7
ATSR1406	Walloon Lake	*P. parkeri*	*T. physellae*	99.9
ATSR1407	*P. parkeri*	*P. parkeri*	*T. physellae*	99.9
ATSR1431	Long Lake	*Gyraulus* sp.	Avian schistosomatid	87
ATSR1443	Elk Lake	*S. emarginata*	*T. stagnicolae*	99.8
ATSR1459	Lake Skegemog	*S. emarginata*	*T. stagnicolae*	99.3
ATSR1460	Lake Skegemog	*S. emarginata*	*T. stagnicolae*	99.2

### 3.2 Species specific qPCR assays

#### 3.2.1 Assay design

Assays were designed to the CO1 region of *T. physellae* (Assay limit of detection 95 (LOD_95_): 12 copies/5 μL)*, T. stagnicolae* (Assay LOD_95_: 27 copies/5 μL)*, T. szidati* (LOD_95_: 7.9 copies/5 μL), and *A. brantae* (LOD_95_: 57.8 copies/5 μL) (Wilrich and Wilrich, 2009) (Table 1, Supplementary Fig. 1). Specificity was assessed *in silico* and experimentally. CO1 regions of each species targeted were aligned against other related avian trematodes species, and diagnostic tests targeted the regions that were most different, in the case of *T. physellae* locked nucleic acids were added to key nucleotides to ensure specificity. Cross reactivity was tested against each *Trichobilharzia* and *Anserobilharzia* species, as well as non-avian trematode species which were selected because they were the most prevalent species identified during our snail survey (Table 3). No cross reactivity was observed.

**Table 3:** **Specificity of the species-specific diagnostic**. Each assay was tested for cross reactivity against purified DNA extracts of other trematode species. *Non-avian schistosome genera tested were: *Australapatemon* sp*., Cotylurus* sp., *Diplostomum* sp., *Notocotylus* sp., *Plagiorchis* sp., *Proterometra* sp., and *Uvulifer* sp.

Assay	Target					
	*T. stagnicolae*	*A. brantae*	*T. szidati*	*T. physellae*	*T. szidati*	Non-avian schistosomes*
*T. stagnicolae*	+	–	–	–	–	–
*A. brantae*	–	+	–	–	–	–
*T. szidati*	–	–	+	–	–	–
*T. physellae*	–	–	–	+	–	–

#### 3.2.2 Water monitoring data

Of the seven lakes studied, four lakes, Lime Lake, Big and Little Glen Lake, and North Lake Leelanau, had merganser relocation programs in 2017 and 2018 in an attempt to control swimmer's itch. South Lake Leelanau, Long Lake, Elk Lake, Skegemog Lake, Lake Charlevoix and Walloon Lake did not have control programs. 25 L water samples were taken mid-July from sites around each lake to assess cercariae concentrations. Elk Lake, Walloon Lake, North Lake Leelanau and Glen Lake had the highest numbers of avian schistosome cercariae per litre (Fig. 1).

**Fig. 2:**
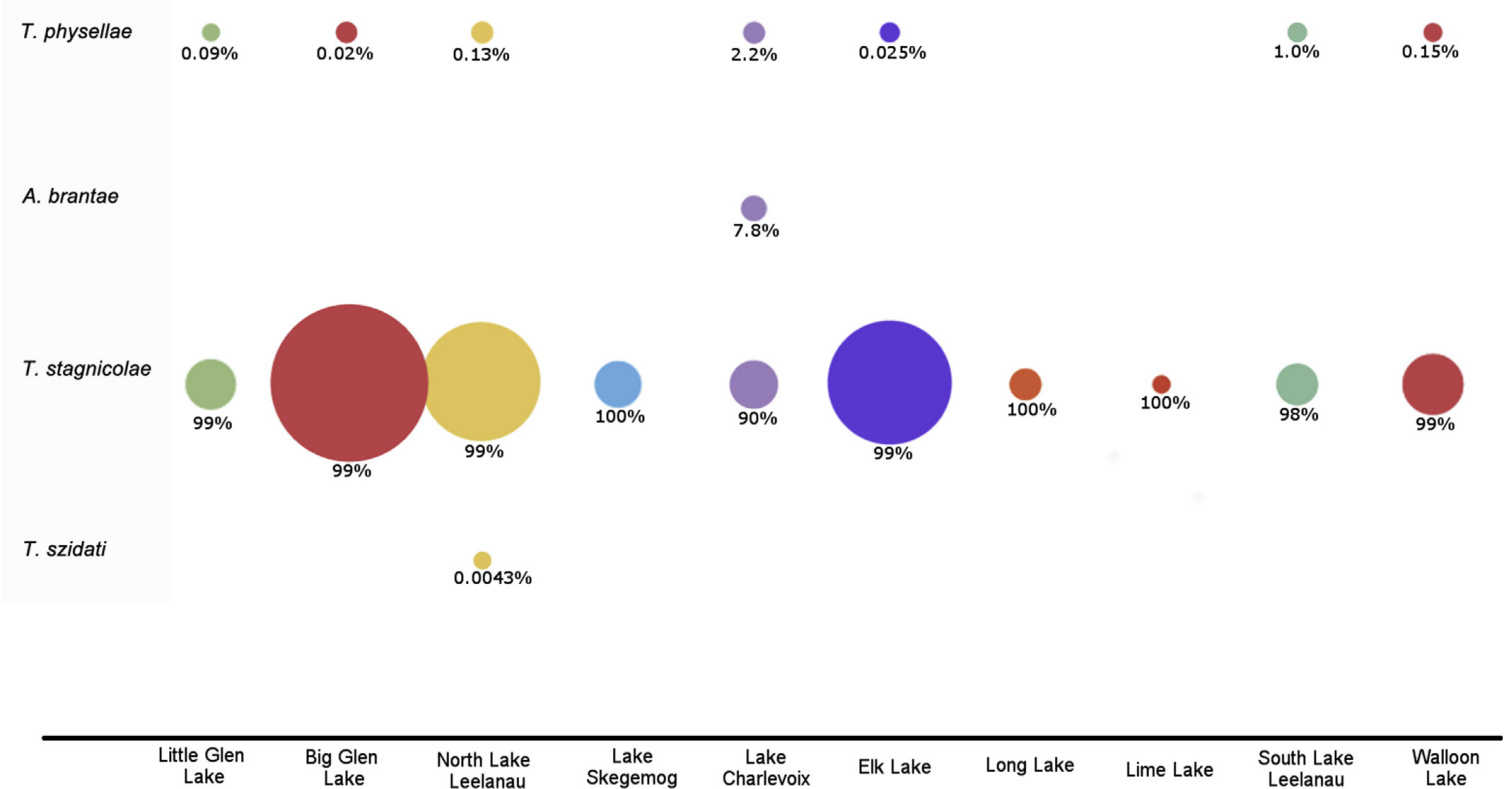
**Percent contribution *of T. stagnicolae, T. szidati, T. physellae* and *A. brantae* species to each lake**. Water samples from different locations and dates were tested using the species-specific qPCR assay and results were pooled by lake to understand the relative contribution overall of each species to each lake. The percent contribution (based on gene copy number) of each species was calculated.

Water samples from each lake were randomly selected to be tested in the species-specific qPCR analyses. Samples that were negative for the 18S avian schistosome assay were excluded. Water samples from different locations and dates were tested using the species-specific qPCR assays and results were pooled by lake to understand the relative contribution overall of each species to each lake. *Trichobilharzia stagnicolae* was the dominant parasite species present in the water samples. However, Big and Little Glen Lake, North Lake Leelanau, Lake Charlevoix, Elk Lake, South Lake Leelanau, and Walloon Lake, also had detectable quantities of *T. physellae*. Lake Charlevoix was the only lake to have detectable amounts of *A. brantae* present in the water, while *T. szidati* was found at North Lake Leelanau (Fig. 2).

**Fig. 3:**
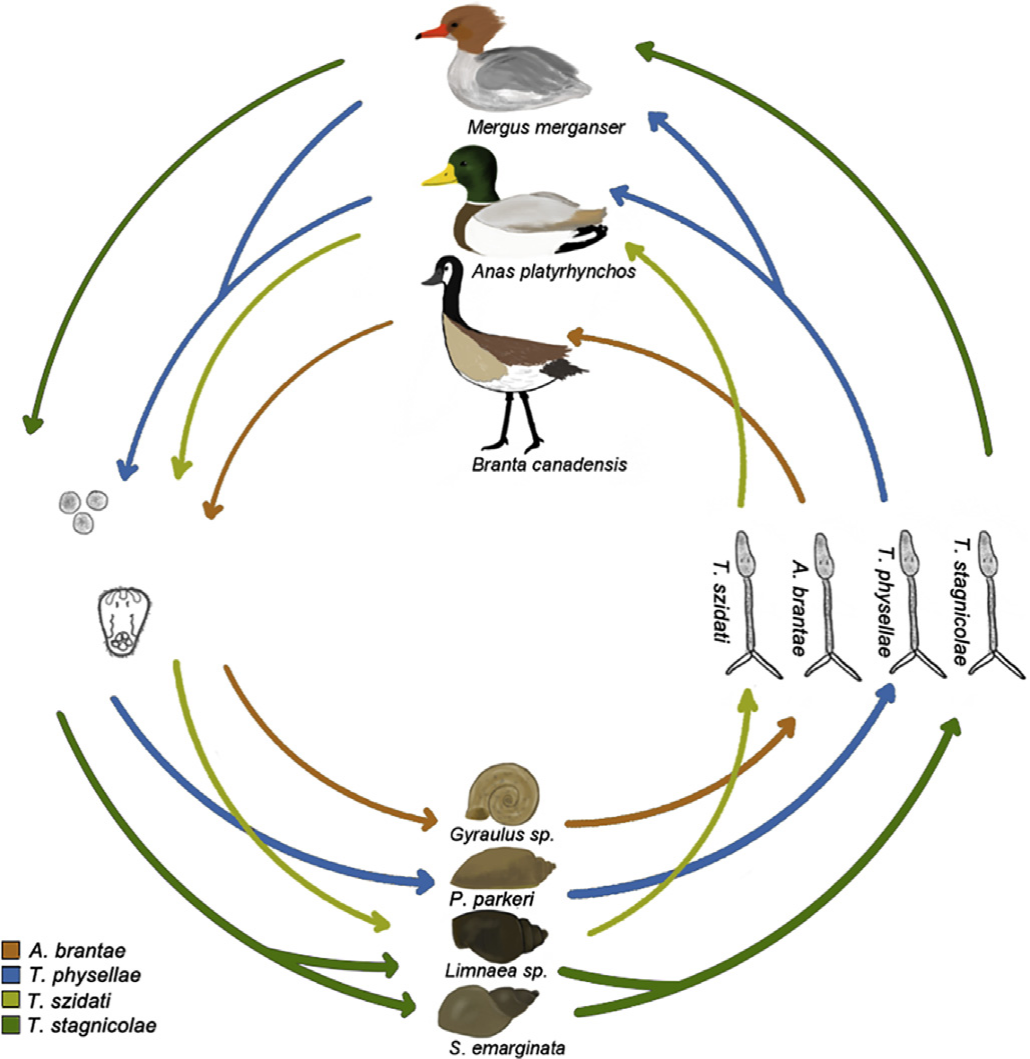
**Lifecycles of *T. stagnicolae, A. brantae, T. szidati,* and *T. physellae***. life cycle summary of the avian schistosome species targeted for species-specific qPCR tests designed in this study.

Snail diversity data collected during the study indicates that *S. emarginata*. and *P. gyrina* snails are present at every lake sampled. Lake Charlevoix, the only lake found to have detectable levels of *A. brantae*, also had *Gyralus* sp. snails, as did Elk, Glen, Long, Lime, Walloon and North Lake Leelanau. Interestingly, we did not find any *Lymnaea* sp. snails in North Lake Leelanau, despite the presence of *T. szidati* in the water. Likely our surveys did not capture *Lymnaea* sp. snails as we had few collection sites on North Lake Leelanau, and we typically find *Lymnaea* sp. snails at low abundance in Northern Michigan (Table 4). Shoreline waterfowl surveys were also conducted for each lake. Mallard ducks were the most abundant bird recorded at all lakes, regardless of whether a merganser control program was in place, followed by Canada geese (Table 5).

**Table 4:** Snail genera observed in each lake.

Snail	Lake								
	Charlevoix	Elk	Glen	South Leelanau	North Leelanau	Lime	Long	Skegemog	Walloon
*S.* emgarinata	**+**	**+**	**+**	**+**	**+**	**+**	**+**	**+**	**+**
*P. parkeri*	**+**	**+**	**+**	**+**	**+**	**+**	**+**	**+**	**+**
*Lymnaea* sp.						**+**			**+**
*Gyraulus* sp.	**+**	**+**	**+**		**+**	**+**	**+**		**+**
*Pleurocera* sp.	**+**	**+**	**+**	**+**	**+**	**+**	**+**	**+**	**+**
*Helisoma* sp. (=*Planorbella* sp.)	**+**	**+**	**+**	**+**	**+**	**+**	**+**	**+**	**+**
*Campeloma decisum*.			**+**	**+**				**+**	**+**
*Marstonia lustrica*	**+**		**+**	**+**	**+**	**+**	**+**	**+**	**+**
*Viviparus* sp.							**+**

**Table 5:** **Waterfowl densities by lake (birds/shoreline mile)**. Complete shoreline waterfowl surveys conducted in mid-July determined the definitive host diversity on each lake. Data are in birds/shoreline mile. Asterisks (*) indicate lakes were common mergansers are actively relocated.

Lake	Bird					
	Mallard	Canada Goose	Mute Swan	Common Merganser	Hooded Merganser	Red Breasted Merganser
Lake Charlevoix	7.08	2.78	0.10	1.12	0.30	0.00
Elk Lake	5.25	1.54	0.14	0.54	0.00	0.21
Big Glen Lake	8.70	0.65	0.37	0.28*	0.00	0.00
Little Glen Lake	9.22	1.25	0.00	4.22*	0.00	0.00
North Lake Leelanau	6.27	0.87	0.40	0.07*	0.00	0.00
South Lake Leelanau	14.12	1.72	0.00	0.00	0.08	0.00
Lime Lake	8.33	0.95	0.00	0.48*	0.00	0.00
Long Lake	6.47	0.66	0.00	0.00	0.06	0.00
Lake Skegemog	2.73	1.20	0.93	0.00	0.00	0.00
Walloon Lake	1.61	1.34	0.00	1.80	0.00	0.00

## 4 Discussion

Results of monitoring for swimmer's itch using the 18S pan-avian schistosome assay demonstrated that swimmers itch continues to be a problem at lakes in Northern Michigan even with continued relocation of common mergansers, with some water samples containing hundreds of cercariae per litre (Fig. 1). As has previously been demonstrated, cercariae move with the bulk flow of water; wind and water movement towards shore concentrate cercariae in different locations (Froelich et al., 2019; Rudko et al., 2018). Snail surveys of avian schistosomes followed by CO1 barcoding identified three species of *Trichobilharzia (szidati, stagnicolae,* and *physellae)*, and *A. brantae,* infecting four species of snail, and three bird hosts at the lakes studied (Fig. 3, Table 2). The presence of other swimmer's itch causing parasites in the area and their hosts may undermine the merganser relocation control effort, as it suggests that the swimmer's itch issues stem from multiple species, rather than just one. This observation prompted us to hypothesize that perhaps one of these other species of swimmer's itch causing avian schistosomes could now be present at a high relative abundance, and be dominating cases of swimmer's itch in the region.

Using these species-differentiating qPCR tests to identify the species contributing to swimmer's itch in our study lakes has yielded a never before seen level of resolution. The results of the species-specific qPCR assays show that while *T. physellae* also cycles at many lakes, they are still observed in far lower abundance than *T. stagnicolae* (Fig. 2), which is contrary to the hypothesis that merganser relocation should be reducing *T. stagnicolae* abundance. Despite the high density of Canada geese present at all the lakes studied, *A. brantae* was only detected at Lake Charlevoix, and, again, despite the large number of mallards observed on the lakes, *T. szidati* was only detected at North Lake Leelanau.

The high relative abundance of *T. stagnicolae* at all of the lakes in question, even those (Big and Little Glen lake, North Lake Leelanau, Lime Lake) that have active control programs suggests that these programs are not effective at interrupting the *T. stagnicolae* life cycle. We hypothesize that either that a second bird host, sympatric with *M. merganser* could also host *T. stagnicolae,* or that the *T. stagnicolae* population is being driven by non-resident merganser populations that are likely comprised of migratory birds in the fall or spring (ie: those that are not targeted by the merganser brood relocation programs). While we have identified in this paper *T. stagnicolae* miracidia in the feces of *M. merganser,* it is possible another host exists that is also contributing *T. stagnicolae* to the ecosystem. *T. stagnicolae* is capable of infecting canaries (Brant and Loker, 2009a,b), and other passerine bird species have also been documented with *Gigantobilharzia* infections, and thus, could also be host to other avian schistosomes such as *T. stagnicolae*. Based on our data, while we could only speculate on other potential hosts, 57 species of bird were observed in Michigan during the North American Breeding Bird Survey (BBS) from 2016 to 2017 alone, with over 25 species of water bird identified (Pardieck et al., 2018). Nonetheless, we are confident based on our survey data that mallards do not transmit *T. stagnicolae* (Table 2), which has also been corroborated experimentally by challenging mallards and Peking ducks (Brant and Loker, 2009a,b). Our previous work has not found *T. stagnicolae* miracidia in the feces of *B. canadiensis* (Rudko et al., 2018); however, their abundance in the region perhaps warrants a more comprehensive assessment of the schistosome species present in their feces (Table 5).

The utility of this work extends far beyond the scope of swimmer's itch monitoring and assessment of trematode flatworm biodiversity. Molecular methods, including qPCR, have been used for decades in public health microbiology to source track contributions of fecal indicator bacteria to different watersheds (Joyce M. Simpson et al., 2002). With this study we aim to demonstrate the utility of such an approach for detecting parasites in water bodies. Many parasites utilize environmental pathways, like water, but also soil or sediments, as a means of transmission, and with more genetic information becoming available, it is possible to achieve species level resolution for certain targets in these matrices. Such assays can answer fundamental questions about parasite distribution in complex systems with regards to environmental variables, but also can provide insight into definitive and intermediate hosts in the area. Moreover, many parasitic flatworms are of agricultural or human health relevance. As control efforts for these parasites places continued pressure on specific life cycles stages, to ultimately achieve control, the life cycle must be broken, which could be assessed using qPCR assays targeting the environmental stages of these parasites.

This study was prompted by the observation, and reports from residents, of the continued issue of swimmer's itch in Michigan. After undertaking a comprehensive survey of parasites found in bird feces and snails, we identified 3 other species of avian trematode present at nine lakes in northern Michigan. We developed highly sensitive and specific diagnostic qPCR tests capable of determining the relative abundance of each species to avian schistosome positive water samples. To our surprise, our results demonstrate that *T. stagnicolae* continues to be the dominant parasite, even at lakes undertaking laborious and expensive merganser relocation programs. There remains much to be discovered regarding the life history of *T. stagnicolae* in Michigan, and future work will continue to investigate if migratory mergansers contribute parasites into the lake in early spring and late fall, resulting in snail infections and swimmer's itch outbreaks during the summertime, or if a sympatric host, yet undiscovered, could be contributing this elusive, yet abundant parasite into the waters of northern Michigan.

## Figures and Tables

**SuppFigure1-2.0:**
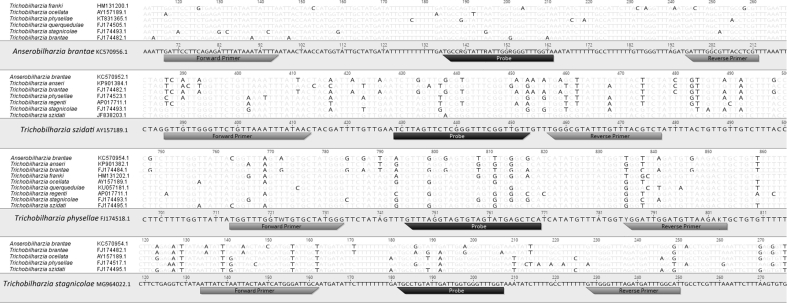
**Primer and probe locations for each assay.** Alignments of the CO1 gene regions with the primer and probe locations for each assay.

## Footnotes

1 Nucleotide sequences reported in this paper have been deposited into Genbank under accession numbers: **MK433243, MK433244, MK433245, MK433246, MK433247, MK433248, MK433249, MK433250, MK433251 MK433252**.
